# Characterization of Primary Cultures of Normal and Neoplastic Canine Melanocytes

**DOI:** 10.3390/ani11030768

**Published:** 2021-03-10

**Authors:** Monica Sforna, Elisabetta Chiaradia, Ilaria Porcellato, Serenella Silvestri, Giulia Moretti, Luca Mechelli, Chiara Brachelente

**Affiliations:** Department of Veterinary Medicine, University of Perugia, 06126 Perugia, Italy; monica.sforna@unipg.it (M.S.); silvestri.serenell@gmail.com (S.S.); giulia-89_@hotmail.it (G.M.); luca.mechelli@unipg.it (L.M.); chiara.brachelente@unipg.it (C.B.)

**Keywords:** melanocytes, mucosal melanoma, cutaneous melanoma, primary cell culture, dog

## Abstract

**Simple Summary:**

Melanoma is one of the most aggressive cancers in humans, with high rates of metastasis and a poor prognosis. Because of its environmental, biological and genetic features, numerous studies indicate the dog as a good comparative model for human melanoma. Primary cell cultures of healthy and neoplastic melanocytes derived from skin and oral mucosa of dogs with spontaneous tumors are established in this study. This model could represent a suitable tool to compare biological and molecular features of normal and neoplastic melanocytes from the same patient, to investigate the pathways underlying the oncogenic transformation, and to apply a more personalized therapeutic strategy. The cell cultures also meet international guidelines that encourage the use of alternative models to animal ones for the study of oncological diseases.

**Abstract:**

Although numerous animal models, especially mouse models, have been established for the study of melanoma, they often fail to accurately describe the mechanisms of human disease because of their anatomic, physiological, and immune differences. The dog, as a spontaneous model of melanoma, is nowadays considered one of the most valid alternatives due to the heterogeneity of clinical presentations and of histological and genetic similarities of canine melanoma with the human counterpart. The aim of the study was to optimize a protocol for the isolation and cultivation of healthy and neoplastic canine melanocytes derived from the same animal and obtained from cutaneous and mucosal (oral) sites. We obtained five primary tumor cell cultures (from 2 cutaneous melanoma, 2 mucosal melanoma and 1 lymph node metastasis) and primary normal melanocyte cell cultures (from normal skin and mucosa) from the same dogs. Immunocytochemical characterization with Melan A, PNL2 and S100 antibodies confirmed the melanocytic origin of the cells. This work contributes to expanding the case record of studies on canine melanoma cell cultures as suitable model to study human and canine melanoma. To the authors’ knowledge, this is the first report of isolation of normal skin and mucosal canine melanocytes.

## 1. Introduction

Despite the continuous scientific effort in the development of new therapeutic strategies, melanoma still represents one of the most aggressive malignancies in humans and dogs, with high rates of metastasis and poor prognosis [1,2,3]. Different animal models are employed by researchers for melanoma studies, in particular genetically engineered mouse models, albeit they revealed some limits and failed to effectively predict the human response due to differences in physiology and immunity [4,5,6]. Numerous studies have highlighted similarities between human and canine melanoma, particularly the mucosal ones, both from a morphological, cytogenetic and molecular point of view, with activation of common pathways. The sharing of the same living environment and, therefore, the possible exposure to the same carcinogens could make the dog an excellent model for the study of spontaneous neoplastic diseases [2,4,7,8]. Canine melanoma, as a model of naturally occurring melanoma, shares with the human counterpart multiple physiopathological mechanisms. In particular, the dog has different clinical presentations of melanoma with different biological behaviors: skin tumors usually have a benign behavior with a good prognosis, differently from what happens in humans for skin melanomas, which are related to UV exposure. On the other hand, mucosal and acral melanomas (melanomas with digital and ungual localization) are very aggressive, with rapid growth and a poor prognosis, both in dogs and humans. These types of canine melanomas represent an excellent comparative model for human non-UV-induced melanomas [9,10].

In addition, international guidelines are increasingly encouraging the development of alternative techniques and processes that contribute to reduce the use of animals for experimental purposes [6]. In this panorama, the use of melanoma cell cultures represents a promising tool, mainly for biological studies and preliminary toxicity and efficacy screening of therapeutic molecules [6]. Commercially available human melanoma cell lines were frequently used in the past to study new target therapies, especially for human cutaneous melanoma, although several problems such as the genomic instability and the possible contamination with other cells over time were encountered [11]. A more modern approach involves the use of primary cultures obtained directly from the patient’s tumor tissue; this tool has the advantage of sharing the same characteristics with the original tumor, allowing to work on the personalization of the patient’s treatment [11]. This approach is even more appropriate for melanocytic tumors that exhibit different biological behavior in relation to the site of onset, in humans as well as in dogs. In veterinary medicine, isolation of canine melanoma cell lines is rarely reported and even rarer is the description of the isolation and cultivation of normal melanocytes, with only one report, to our knowledge, describing the isolation of normal uveal melanocytes [1,12,13,14]. Furthermore, for dogs, the use of primary cell cultures for the study of melanoma is still a very important tool, as there are very few stabilized commercial cell lines of canine melanoma [15].

The aim of this study was to standardize a method of isolation and culture of normal and neoplastic melanocytes derived from canine cutaneous and mucosal tissues and from metastatic melanoma. This would represent a suitable tool to compare biological and molecular features of normal and neoplastic cells from the same patient, to investigate the pathways underlying the oncogenic transformation, and make a further step towards personalized therapeutic strategies.

## 2. Materials and Methods

### 2.1. Sample Collection

Canine melanocytic neoplasms from cutaneous and mucosal sites and their surrounding healthy tissue were aseptically collected and processed within 2 h from surgical excision performed by surgeons of the University of Perugia Veterinary Teaching Hospital or by DVM practitioners. Inclusion criteria for the study was a previous histologic (incisional biopsy) or cytologic diagnosis of melanocytic tumor. For the isolation of normal melanocyte cultures, an additional inclusion criterion was that samples had to have a margin of healthy tissue (skin or mucosa) of at least 1 cm from the tumor, as detected from the macroscopic examination. Upon collection, normal tissue surrounding cutaneous or oral tumors was separated with the aid of scalpel blades, under sterile conditions. Healthy tissue was placed in Dulbecco’s phosphate-buffered saline (DPBS) without Ca^2+^ and Mg^2+^, containing 10 µL/mL of penicillin-streptomycin solution (DPBS-P/S) (Corning Incorporated Life Sciences, Tewksbury, MA, USA), 250 μg/mL of amphotericin B (Sigma-Aldrich, St. Louis, MO, USA) and 100 µg/mL of gentamicin (Sigma-Aldrich) until processing (within 2 h) and kept at 4 °C. Tumor tissue was divided into two parts. One part was placed in modified Dulbecco’s medium, as previously described for normal healthy tissue. The second part was placed in neutral buffered formalin for histological and immunohistochemical investigations, according to the recent literature [16]. Presence or absence of metastases was assessed at the time of surgical excision and, if present, metastasis was also collected and processed for histological examination and cell culture. The study was conducted according to the guidelines of the Declaration of Helsinki, and approved by the Ethics Committee of the University of Perugia (2021-USDPAMM-0041730; 18 February 2021).

### 2.2. Primary Culture of Normal Melanocytes

Samples were washed in DPBS and dissected in small stripes (0.5 mm thick), avoiding areas of ulceration and/or hemorrhage; subcutaneous/submucosal tissue, deep dermis (or *lamina propria* for the mucosa), blood clots and vessels, were removed as much as possible with the use of a magnifying glass. Obtained stripes of tumor tissue were incubated overnight in 2.5 mg/mL of dispase II (Sigma-Aldrich) in DPBS-P/S at 4 °C and placed on a rocking platform. Then, the epidermal/mucosal epithelium layer was separated from the underlying layers with forceps and scalpels and the remaining tissue was placed in trypsin-EDTA solution (Sigma-Aldrich) for 30 min at 37 °C.

The digestion was stopped by adding equine serum (Sigma-Aldrich) [17]. Melanocytes were collected using 100 µm cell strainer (Corning Incorporated Life Sciences), centrifuged twice for 6 min at 400× *g* and resuspended in M254 CF medium (Invitrogen, Carlsbad, CA, USA) supplemented with 1% (*v*/*v*) of Human Melanocyte Growth supplement-2 (HMGS2) (Invitrogen), 10 µL/mL of penicillin-streptomycin solution, 10 µL/mL of amphotericin B, 50 µg/mL of gentamycin, 0.1 µg/mL of cholera toxin (Sigma-Aldrich) and 50 ng/mL of phorbol 12-myristate 13-acetate (TPA) (Sigma-Aldrich). The cells were seeded into 25 cm^2^ flasks at a density of 5 × 10^5^ cells/cm^2^ and then incubated at 37 °C in a humidified atmosphere of 5% CO_2_. The culture medium was changed every 48 h.

### 2.3. Primary Culture of Neoplastic Melanocytes

The samples derived from melanocytic neoplasms (cutaneous, mucosal primary tumors, and metastatic lymph node) were processed following two isolation protocols: spontaneous migration (Section 2.3.1) and enzymatic digestion (Section 2.3.2).

#### 2.3.1. Spontaneous Migration

A fragment of biopsy from each tumor was minced in pieces of 1 mm^3^ under sterile conditions, after carefully removing the necrotic and vascularized areas of the tissue. Fragments were placed in a six-well plates containing Dulbecco’s Modification of Eagle’s Medium (DMEM/F12) (Corning Incorporated Life Sciences) supplemented with 10 µL/mL of penicillin-streptomycin solution, 10 µL/mL of amphotericin B, 50 µg/mL of gentamycin, 5% equine serum (Sigma-Aldrich), 0.5 µg/mL of hydrocortisone (Sigma-Aldrich), 10 µg/mL of insulin (Sigma-Aldrich), 20 ng/mL of epidermal growth factor (EGF) (Corning Incorporated Life Sciences) and incubated at 37 °C in a humidified atmosphere of 5% CO_2_. The medium was changed every 48 h and larger tissue fragments were removed after observation of migrated cells adhering to the plate.

#### 2.3.2. Enzymatic Digestion

The tissue fragments of 0.5 mm of thickness, prepared as previously described for normal melanocytes, were digested with 1.5 mg/mL collagenase II (Sigma-Aldrich) for 2 h at 37 °C. Undigested tissue was removed using a 100 µm cell strainer. Cells were collected by centrifugation (10 min at 400× *g*), and the pellet was resuspended in DMEM/F12 medium supplemented as described for cells obtained by spontaneous migration. The cells were seeded at a density of 2 × 10^4^ cells/cm^2^ and were then incubated at 37 °C in a humidified atmosphere of 5% CO_2_. Culture medium was changed every 48 h.

At third passage, cells were cryopreserved in 15% horse serum and 10% dimethyl sulfoxide (DMSO).

### 2.4. Histochemistry and Immunocytochemistry

Morphological, functional and phenotypical characterization of cells were assayed using hematoxylin and eosin (H&E) staining, DOPA staining and immunocytochemistry, respectively. Briefly, cells were seeded on glass coverslips at a density of 2 × 10^4^ cells/cm^2^ in 24-well plates and incubated at 37 °C in a humidified atmosphere of 5% CO_2_ to about 50% confluence. The medium was then removed, and cells were washed twice in phosphate-buffered saline (PBS), fixed for 15 min at room temperature with 4% paraformaldehyde (pH 7.2), and finally washed with PBS.

DOPA staining was performed incubating cells with 0.1% DOPA (3,4-dihydroxyphenylalianine) (Sigma-Aldrich) at 37 °C for 4 h [18]. Subsequently, nuclear fast red was used as counterstaining.

For immunocytochemistry (ICC), protein block was performed for 1 h at room temperature using a solution containing DPBS, 1% fetal bovine serum, and 0.3% Triton X-100. Phenotypical characterization of melanocytes was performed using antibodies against Melan A (1:150, A103-M27C10-M29E3; Abcam, Cambridge, UK), melanoma-associated antigen (PNL2) (1:150, sc-59306; Santa Cruz Biotechnology, Santa Cruz, CA, USA) and S100 (GA504 1:200; Dako, Denmark). After 1 h of incubation with primary antibodies at room temperature, cells were washed and incubated for 10 min with secondary biotinylated goat anti-polyvalent antibody from a commercially available kit (Mouse and Rabbit Specific HRP Detection IHC Kit, ab93677; Abcam). The immune complexes were revealed by incubating the cells with peroxidase-labelled streptavidin for 10 min and using 3-amino-9-ethylcarbazole (AEC Substrate System, ab64252; Abcam) as chromogen. Carazzi’s hematoxylin staining was used as a counterstain. Cells were observed both with light and phase contrast microscope, after mounting coverslips on glass slides with an aqueous medium agent (Aquatex, 1085620050; Merck KGaA, Darmstadt, Germany).

## 3. Results

### 3.1. Sample Population and Histopathology

**Figure 1:**
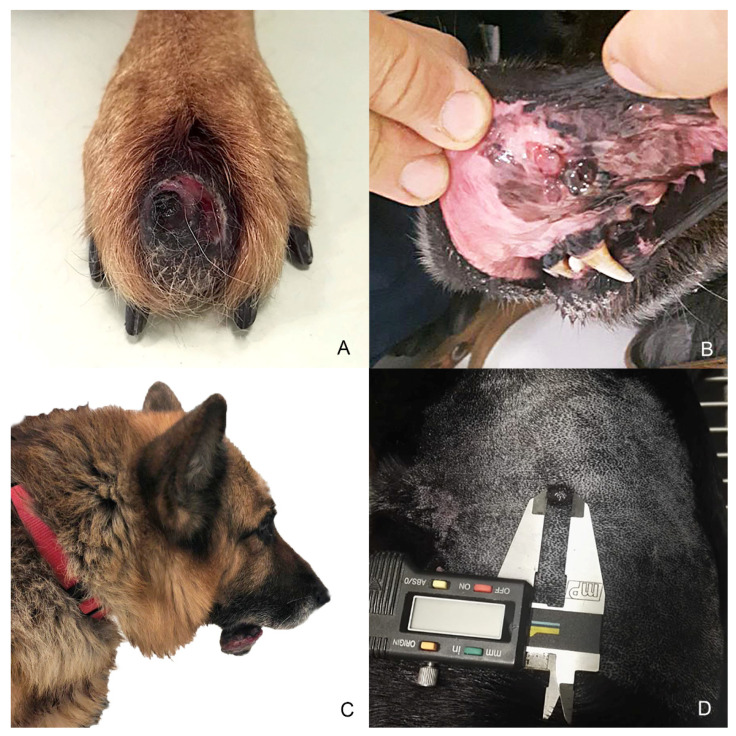
Anatomical localization of selected melanocytic tumors. (**A**) Case n. 1—Interdigital cutaneous melanocytoma; (**B**) Case n. 2—Mucosal (buccal) melanoma; (**C**) Case n. 3—Melanoma arising from the mucous side of the lower lip with lymph node metastases; (**D**) Case n. 4—Cutaneous melanoma of 1 cm of diameter on the left flank area.

**Figure 2:**
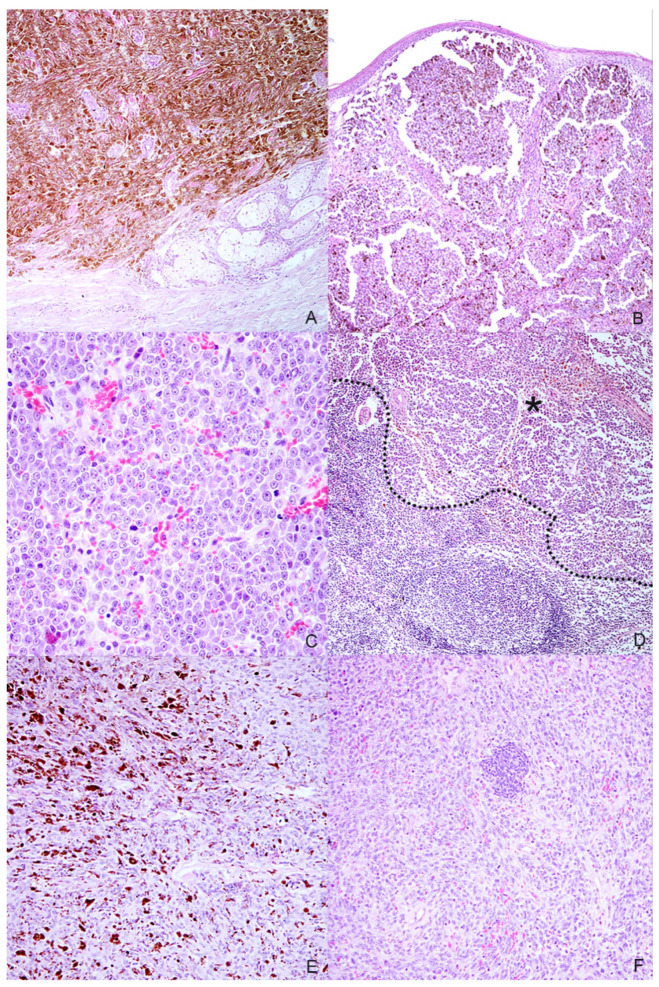
Histopathologic characteristics of the selected melanocytic tumors. (**A**) Case n. 1—Dermal highly cellular proliferation of pigmented melanocytes with minimal cellular atypia (H-E, 100×); (**B**) Case n. 2—Densely packed, moderately pigmented melanocyte proliferation infiltrating the lamina propria of the oral mucosa(H-E, 100×); (**C**) Case n. 3—Mucosal amelanotic melanoma: cells show large nuclei with prominent nucleoli without intracytoplasmic melanin. Note the high mitoses (H-E, 400×); (**D**) Case n. 3—Lymph node metastasis: the same atypical melanocytic population effacing the architecture of the lymph node (residual cortical tissue on the bottom-left of the dotted line; metastatic tumoral tissue on the asterisk) (H-E, 100×); (**E**) Case n. 4—Spindle-shaped, frequently pigmented melanocytes proliferation with moderate cellular atypia, localized in the dermis (H-E, 200×); (**F**) Case n. 5—Mucosal amelanotic melanoma: epithelioid to polygonal neoplastic melanocytes forming sheets and rare nests in the lamina propria (H-E, 200×).

Eight samples were initially collected; however, three cases were excluded during processing because the subsequent histological and immunohistochemical investigations did not confirm the melanocytic origin of the tumor, diagnosed cytologically. Anamnestic data and histological classification of the selected five cases are reported in Table 1. Of the 5 cases, 2 were mixed breed and other 3 dogs were purebred dogs (English setter, German shepherd and Dobermann); 2 dogs were male and 3 female. Cutaneous tumors were localized in the interdigital space and flank, while mucosal ones originated from different sites of the oral cavity and the labial mucosa (Figure 1). Histology supported the melanocytic origin of the tumors and 1 cutaneous melanocytoma, 1 cutaneous melanoma and 3 mucosal melanoma were identified (Figure 2). Immunohistochemical investigation with antibodies against Melan A and PNL2 confirmed the morphological diagnosis in formalin-fixed, paraffin-embedded tissues. At the time of surgical excision of the tumor, only one dog with a mucosal melanoma (Case n. 3) had metastases to the mandibular and retropharyngeal lymph nodes.

### 3.2. Primary Culture of Normal Melanocytes

**Figure 3:**
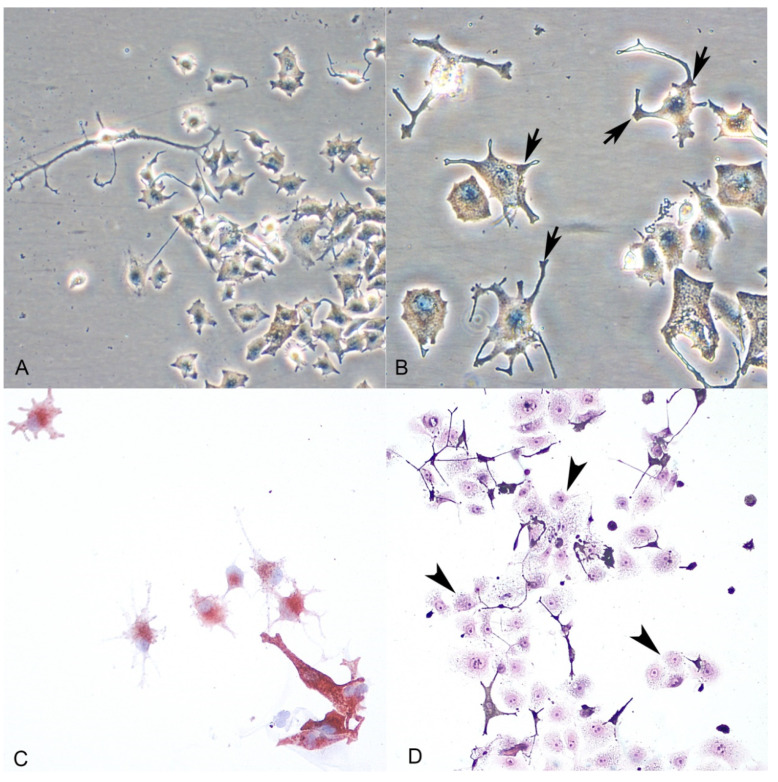
(**A**,**B**): Normal melanocytes (phase-contrast microscopy). Melanocytes show dendritic morphology and the presence of granules of melanin (arrows) within the cytoplasm. (**C**): Cytoplasmic immunolabeling of the cells with the anti-Melan A antibody confirmed melanocytic origin. (**D**): Keratinocyte contamination during the first days of culture (arrowheads) (H-E stain).

Primary cell cultures of normal melanocytes were obtained from 3 out of the 5 cases included in the study. For two dogs, we received incisional biopsies without healthy tissue surrounding the tumors. Melanocytes were adherent to the bottom surface of the culture flask in 24–48 h; cells derived from mucosal and skin samples were polyhedral or elongated in shape with central oval to round nucleus and moderate to abundant cytoplasm, which frequently showed multiple prolongations of different lengths (Figure 3A). Fine and dark granules were observed, frequently located at the end of these multiple dendrites or scattered in the cytoplasm (Figure 3B). During the first days of growth, simultaneous proliferation of keratinocytes (Figure 3D) was seen; the use of TPA (phorbol ester 12-O-tetradecanoylphorbol-13-acetate) as a molecule inhibiting the growth of keratinocytes proved necessary to obtain a pure melanocyte culture. No fibroblast contamination of the melanocytes cultures was seen.

### 3.3. Primary Culture of Neoplastic Melanocytes

**Figure 4:**
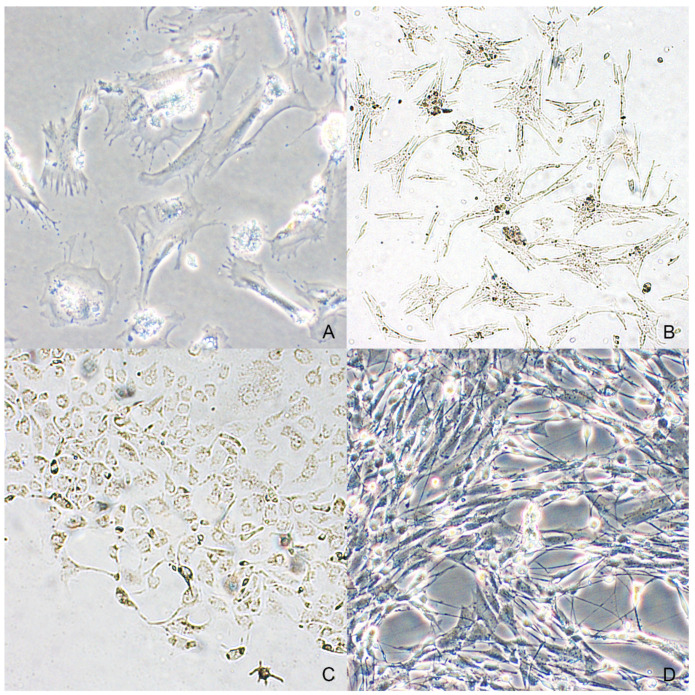
Dendritic to spindle shape was observed in neoplastic melanocytes from an oral melanoma (case n. 2) both in cultures treated with enzymatic digestion (**A**) and in the ones provided by spontaneous migration (**B**). (**C**) Most neoplastic melanocytes from cutaneous melanoma were characterized by a polygonal shape and abundant intracytoplasmic melanin (case n. 4). (**D**) Neoplastic melanocytes from the nodal metastasis of the case n. 3 (phase-contrast microscopy).

Primary cell cultures of neoplastic melanocytes were isolated from all selected cases. Viable and adherent cells were obtained in 24–48 h from both spontaneous migration and enzymatic digestion of the tissue fragments, but none of the cultures obtained by the first method (spontaneous migration) yielded a monolayer of confluent cells (Figure 4A,B). Initially, both types of cell cultures contained a variable amount of dark brown pigment in the medium, which was lost with subsequent changes of the medium. The cells showed predominantly a polyhedral shape with multi-dendritic appearance and sometimes were more elongate with spindle-shaped morphology (Figure 4C). In particular, cells obtained from the lymph node metastasis were spindle with oval to elongated nucleus and presence of cytoplasmic pigment granules; cytoplasmic granules were detected in 3 out of 5 cell cultures obtained from melanocytic tumors (Figure 4D).

### 3.4. Cell Cultures Characterization

Hematoxylin-eosin staining was employed to evaluate in detail the morphologic features of cultured melanocytes. To confirm the production of tyrosinase, an enzyme necessary for the synthesis of melanin, DOPA staining was also performed with a positive reaction. Immunocytochemical expression of Melan-A, PNL2 and S100 antigens were observed in both normal (Figure 3C) and neoplastic melanocytes.

## 4. Discussion

In this study we obtained primary cell cultures of healthy and neoplastic canine melanocytes from different tissues of the same patient. Normal melanocytes were isolated from healthy skin and oral mucosa and neoplastic melanocytes were cultured from cutaneous and oral primary melanocytic tumors, as well as from a metastatic lymph node. The selection criterion of sampling “normal” tissue at least 1 cm away from the tumor allows us to be reasonably confident about the absence of neoplastic cells in that site. Cultured cells from different anatomic locations all showed morphological, functional (DOPA) and immunocytochemical (Melan A, PNL2 and S100) features typically expressed by melanocytes. Literature about canine melanoma cell lines is fairly poor [1,12,19]; the establishment of canine melanoma cell lines represents a further opportunity in the study of melanoma. Indeed, this in vitro tool could provide support to the animal models (mainly mouse models) already widely used for human melanoma, which have the disadvantage of not perfectly replicating the biological behavior of the spontaneous tumor [20]. The canine animal model shares with human melanoma the disease occurrence rate, the aggressiveness, the tendency to metastasize and the difficulty to treat metastases. Molecular and signalling pathways similarities have also been identified [1,5,13].

Immortalized cell lines represent a cost-effective and stable platform that is easy to manipulate and expand, which is very useful for those studies that require high levels of reproducibility [21]. Cell lines have been used for many years in vaccine production, testing drug metabolism and cytotoxicity, antibody production, study of gene function, and synthesis of biological compounds; however, most of them may differ genetically and phenotypically from their tissue origin and also show altered morphology as they are grown over a long period of time [22,23,24]. The type of cell culture chosen depends strictly on the purpose of the research; in the panorama of personalized medicine primary cells retain the characteristic of donor tissue with phenotypic and genetic stability for a limited number of passages. Moreover, primary cells derived from the same species overcome the limits of the use of heterologous tumor cell lines. This is particular important in the dog as very few established and commercial melanoma cell lines are available. In our study, primary cultures were obtained from primary tumors and from one metastatic tumor; the melanocytic origin of malignant cells was confirmed in all cell lines. The availability of cultures from the primary tumor and its metastasis could provide information on changes in terms of clinic-pathological markers and gene signatures in metastases compared to their primary tumors and whether these changes can be significantly associated with prognosis.

In this study, we were able to isolate neoplastic melanocytes from different anatomic sites and matched normal melanocytes from the same animal. This approach would permit the investigation of the main molecular pathways and candidate genes activated in tumoral melanocytes when compared to normal melanocytes. After validation, this model of melanoma primary cells could pave the way to the exploration of new prognostic factors and potentially new specific therapies at a personalized (individual-based) level. Indeed, patient-derived primary culture cells, and in particular organoids, represent an in vitro model appropriate to study cancer heterogeneity. Obtaining specific therapy for the individual’s condition is the goal, in human medicine, of precision and personalized medicine (PPM) through the study of the specific characteristics of the individual patient’s tumor [23]. Primary cell cultures can be employed to acquire omics data (transcriptomic, proteomic, and metabolomic) regarding primary tumors and metastases, necessary to establish tailored therapy for the tumor and patient [23]. Primary cell lines of normal melanocytes have been obtained in humans, mouse, pig, arctic fox but not in dog, with the exception of a single case of isolation of canine uveal melanocytes [5,14,18,25,26,27,28]. In particular, isolation of healthy melanocytes is difficult to achieve since contamination by epithelial cells and fibroblasts, which are more numerous in skin and mucosa and have a much faster growth rate, is common [25]. Melanocytes, indeed, represent about 1 cell for every 10–20 keratinocytes of the basal layer in dog skin but still no data are present on the number of melanocytes in canine oral mucosa [29]. Our experience confirms the problem of keratinocytes contamination of the culture, which we overcame with the use of TPA, a molecule that shows toxic activity towards epithelial cells and is recognized as a promoter of melanocyte growth [25,30]. In our study, we did not encounter significant difficulties with fibroblast contamination, and cell culture characterization confirmed the isolation of pure melanocytes. Moreover, cells isolated from the normal mucosa of the oral cavity are also associated with a microenvironment that is normally contaminated by the oral microbial flora. For these cases, a double concentration of antibiotic was employed for the first two changes of the culture medium.

For the isolation of neoplastic melanocytes, we tested two types of protocols derived from the scientific literature: spontaneous migration and enzymatic digestion. We obtained positive results in particular by enzymatic digestion of the tumors and subsequent incubation in flasks, while through spontaneous migration, the cells showed early detachment or never reached the confluence to the culture plates. However, when the isolation was effective, no morphological differences were observed between the melanocytes obtained with the two methods. Scientific reports on the isolation and cultivation of both canine and human neoplastic melanocytes, regardless of the technique used (spontaneous migration or enzymatic digestion), frequently favor a flask incubation of the cells, although occasionally plate seeding is described [1,11,12,19,31]. Accordingly, we preferred the enzymatic digestion as a larger number of cells was obtained in less time.

A notable limitation of the study is related to the diagnosis of melanocytic tumors necessary before the surgical excision and the subsequent starting of the isolation protocol for neoplastic melanocytes. An incisional biopsy prior to complete excision of the tumor would be the ideal procedure to guide the selection of tissue to culture; however, several factors related to owners’ decisions or patient-related factors can make this step difficult. A suspicion of melanocytic neoplasm can emerge from the cytological examination of the mass, which is frequently performed being less invasive and easier to perform compared to a biopsy. However, melanocytic tumors, and especially oral melanomas, are often poorly differentiated neoplasms with marked cellular atypia and lack of melanin pigment (amelanotic melanomas). The lack of typical cytological features of melanocytic origin lead then to a list of possible differential diagnosis including other neoplasms such as sarcomas and poorly differentiated carcinomas; the same diagnostic dilemma sometimes can occur in histological samples. This limit could be overcome using immunohistochemistry or immunocytochemistry investigations, respectively, on incisional biopsies or fine-needle aspirates (FNA) of the mass, but excisional surgery often takes place without a definitive diagnosis. In our study, the isolation protocol of neoplastic cells in three cases had to be aborted upon initiation because the initial temptative histologic diagnosis of oral melanoma was ruled out after immunohistochemical characterization. In a cytologically suspected case of melanoma included in this study, the diagnosis was confirmed by the authors through the use of immunocytochemistry with anti-Melan A and anti PNL2 markers on FNA of the oral mass. The accuracy of this technique in our experience was very good, as confirmed by the literature [32,33]. Our study is a preliminary work, and next steps will be the correct validation of the proposed model that it could represent a support and an additional tool for the use of the dog as a natural model to investigate melanoma development and progression.

## 5. Conclusions

To the authors’ knowledge, this is the first report of isolation of normal skin and mucosal canine melanocytes from the same patients. This work can contribute to expand the case record of studies on melanoma cell cultures and highlights the importance of multiple models to be integrated with each other in the study of canine and human melanoma. Studies on the genetics of melanocytes in tumorigenesis and on the efficacy of therapeutic molecules with an increasingly requested approach of personalized medicine to the patient can be performed. Ongoing and future studies are aimed at the realization of a three-dimensional cell culture model of melanocytic tumors to increase the potential of the 2D-model.

## Figures and Tables

**Table 1:** Anamnestic data and histologic diagnosis of selected cases of canine melanocytic tumors. CMC = cutaneous melanocytoma; MM = mucosal melanoma; CM = cutaneous melanoma; NA = Not Available (only the neoplastic cells were cultured). ^§^ Dimensions = range of the minimum and maximum distance of the margins from the tumor. Area = cutaneous or mucosal surface used for normal tissue culture. Distance = centimeters from the tumor where the sample for normal tissue culture was taken.

Case	Dog			Tumor			Margins ^§^		
	Breed	Age	Sex	Primary Site	Distant Metastases	Histologic Diagnosis	Dimensions (Range in cm)	Area (cm^2^)	Distance (cm)
1	Mixed breed	8	M	Skin (interdigital)	No	CMC	1.2–2	2	1.5
2	English setter	11	F	Buccal mucosa	No	MM	0.5–2	0.5	1
3	German shepherd	10	M	SLabial mucosa	Yes	MM	NA	NA	NA
4	Dobermann	9	F	Skin-flank	No	CM	1–2.6	1	1.5
5	Mixed breed	14	F	Oral cavity	No	MM	NA	NA	NA

## Data Availability

The data presented in this study are available on request from the corresponding author.

## References

[1] Segaoula Z., Primot A., Lepretre F., Hedan B., Bouchaert E., Minier K., Marescaux L., Serres F., Galiègue-Zouitina S., André C. (2018). Isolation and characterization of two canine melanoma cell lines: New models for comparative oncology. BMC Cancer.

[2] Nishiya A.T., Massoco C.O., Felizzola C.R., Perlmann E., Batschinski K., Tedardi M.V., Garcia J.S., Mendonça P.P., Teixeira T.F., Zaidan Dagli M.L. (2016). Comparative aspects of canine melanoma. Vet. Sci..

[3] Porcellato I., Brachelente C., De Paolis L., Menchetti L., Silvestri S., Sforna M., Vichi G., Iussich S., Mechelli L. (2019). FoxP3 and IDO in Canine Melanocytic Tumors. Vet. Pathol..

[4] Prouteau A., André C. (2019). Canine melanomas as models for human melanomas: Clinical, histological, and genetic comparison. Genes.

[5] Aktary Z., McMahon M., Larue L. (2018). Animal Models of Melanoma. Melanoma.

[6] Marconi A., Quadri M., Saltari A., Pincelli C. (2018). Progress in melanoma modelling in vitro. Exp. Dermatol..

[7] Hernandez B., Adissu H.A., Wei B.R., Michael H.T., Merlino G., Simpson R.M. (2018). Naturally occurring canine melanoma as a predictive comparative oncology model for human mucosal and other triple wild-type melanomas. Int. J. Mol. Sci..

[8] Tarone L., Barutello G., Iussich S., Giacobino D., Quaglino E., Buracco P., Cavallo F., Riccardo F. (2019). Naturally occurring cancers in pet dogs as pre-clinical models for cancer immunotherapy. Cancer Immunol. Immunother..

[9] Gillard M., Cadieu E., De Brito C., Abadie J., Vergier B., Devauchelle P., Degorce F., Dreano S., Primot A., Dorso L. (2014). Naturally occurring melanomas in dogs as models for non-UV pathways of human melanomas. Pigment Cell Melanoma Res..

[10] Simpson R.M., Bastian B.C., Michael H.T., Webster J.D., Prasad M.L., Conway C.M., Prieto V.M., Gary J.M., Goldschmidt M.H., Esplin D.G. (2014). Sporadic naturally occurring melanoma in dogs as a preclinical model for human melanoma. Pigment Cell Melanoma Res..

[11] Welte Y., Davies C., Schäfer R., Regenbrecht C.R.A. (2013). Patient derived cell culture and isolation of CD133+ putative cancer stem cells from melanoma. J. Vis. Exp..

[12] Inoue K., Ohashi E., Kadosawa T., Hong S.H., Matsunaga S., Mochizuki M., Nishimura R., Sasaki N. (2004). Establishment and Characterization of Four Canine Melanoma Cell Lines. J. Vet. Med. Sci.

[13] Touil Y., Segaoula Z., Thuru X., Galiegue-Zouitina S., Tierny D., Quesnel B. (2020). Aggressiveness Potential of Spontaneous Canine Mucosal Melanoma Can Dictate Distinct Cancer Stem Cell Compartment Behaviors in Regard to Their Initial Size and Expansion Abilities. Stem Cells Dev..

[14] Dawson-Baglien E.M., Winkler P.A., Bruewer A.R., Petersen-Jones S.M., Bartoe J.T. (2015). Isolation and cultivation of canine uveal melanocytes. Vet. Ophthalmol..

[15] Lin W., Modiano J.F., Ito D. (2017). Stage-specific embryonic antigen: Determining expression in canine glioblastoma, melanoma, and mammary cancer cells. J. Vet. Sci..

[16] Smedley R.C., Spangler W.L., Esplin D.G., Kitchell B.E., Bergman P.J., Ho H.-Y., Bergin I.L., Kiupel M. (2011). Prognostic markers for canine melanocytic neoplasms: A comparative review of the literature and goals for future investigation. Vet. Pathol..

[17] Wessels D., Lusche D.F., Voss E., Kuhl S., Buchele E.C., Klemme M.R., Russell K.B., Ambrose J., Soll B.A., Bossler A. (2017). Melanoma cells undergo aggressive coalescence in a 3D Matrigel model that is repressed byanti-CD44. PLoS ONE.

[18] Wang D., Xu X., Ma H., Yue X., Li C., Zhu W. (2013). Optimization of the method for the culture of melanocyte precursors from hair follicles and their activation by 1,25-dihydroxyvitamin D3. Exp. Ther. Med..

[19] Lourenço S.V., Bologna S.B., Hsieh R., Sangueza M., Fernandes J.D., Nico M. (2013). Establishment and characterization of an oral mucosal melanoma cell line (MEMO) derived from a longstanding primary oral melanoma. Am. J. Dermatopathol..

[20] Van der Weyden L., Brenn T., Patton E.E., Wood G.A., Adams D.J. (2020). Spontaneously occurring melanoma in animals and their relevance to human melanoma. J. Pathol..

[21] Esparza-López J., Martínez-Aguilar J.F., de Ibarra-Sánchez M.J. (2019). Deriving Primary Cancer Cell Cultures for Personalized Therapy. Rev. Investig. Clin..

[22] Pastor D.M., Poritz L.S., Olson T.L., Kline C.L., Harris L.R., Koltun W.A., Chinchilli V.M., Irby R.B. (2010). Primary cell lines: False representation or model system? A comparison of four human colorectal tumors and their coordinately established cell lines. Int. J. Clin. Exp. Med..

[23] Lin J.J. (2019). Cancer Treatment. Caring Patients Across Cancer Care Contin. Essent. Prim. Care.

[24] Kaur G., Dufour J.M. (2012). Cell lines. Spermatogenesis.

[25] Tang J., Li Q., Cheng B., Jing L. (2014). Primary culture of human face skin melanocytes for the study of hyperpigmentation. Cytotechnology.

[26] Julé S., Bossé P., Egidy G., Panthier J.J. (2003). Establishment and characterization of a normal melanocyte cell line derived from pig skin. Pigment Cell Res..

[27] Bao J., Wang L., Wang G., Liu X., Yang F. (2015). Isolation and culture of melanocytes from the arctic fox (*Alopex lagopus*). Ital. J. Anim. Sci..

[28] Dagnino L., Crawford M. (2018). Isolation, Culture, and Motility Measurements of Epidermal Melanocytes from GFP-Expressing Reporter Mice. Skin Stem Cells Methods Mol. Biol..

[29] Miller W., Griffin C.E., Campbell K.L. (2013). Muller and Kirk’s Small Animal Dermatology.

[30] Scott G. (2014). Selective proliferation of normal human melanocytes in vitro in the presence of phorbol ester and cholera toxin by eisinger and marko. Exp. Dermatol..

[31] Wood E.A., Lu Z., Jia S., Assumpção A.L.F.V., Van Hesteren M.A., Huelsmeyer M.K., Vail D.M., Pan X. (2020). Pevonedistat targeted therapy inhibits canine melanoma cell growth through induction of DNA re-replication and senescence. Vet. Comp. Oncol..

[32] Przeździecki R., Czopowicz M., Sapierzyński R. (2015). Accuracy of routine cytology and immunocytochemistry in preoperative diagnosis of oral amelanotic melanomas in dogs. Vet. Clin. Pathol..

[33] Höinghaus R., Mischke R., Hewicker-Trautwein M. (2002). Use of immunocytochemical techniques in canine melanoma. J. Vet. Med. Ser. A Physiol. Pathol. Clin. Med..

